# Characteristics of diffuse large B‐cell lymphoma in patients with primary Sjögren's syndrome

**DOI:** 10.1111/1756-185X.13800

**Published:** 2020-02-25

**Authors:** Vadim Romanovich Gorodetskiy, Natalya Alexandrovna Probatova, Vladimir Ivanovich Vasilyev

**Affiliations:** ^1^ Department of Intensive Methods of Therapy V.A. Nasonova Research Institute of Rheumatology Moscow Russia; ^2^ Department of Pathology N.N. Blokhin Russian Cancer Research Center Moscow Russia

**Keywords:** clinicopathological findings, diffuse large B‐cell lymphoma, primary Sjögren's syndrome, prognostication, subtyping

## Abstract

**Aim:**

Patients with primary Sjögren's syndrome (pSS) have an increased risk of developing diffuse large B‐cell lymphoma (DLBCL), which is an aggressive and heterogeneous non‐Hodgkin lymphoma. This study aimed to characterize DLBCLs in patients with pSS.

**Method:**

We identified 18 patients with DLBCL and pSS over a 22‐year period. Based on the 2016 WHO guidelines, we characterized DLBCL based on immunohistochemical tests using a broad panel of antibodies, and an Epstein‐Barr virus (EBV) test using in situ hybridization.

**Results:**

The median time from initial pSS symptom onset to the DLBCL diagnosis was 20.5 years and the median time from the pSS diagnosis until the DLBCL diagnosis was 14 years. After the lymphoma diagnosis, the median overall survival was 3 months (range: 0‐212 months) and the 5‐year overall survival rate was 37.5%. Thirteen DLBCLs were re‐classified as DLBCL, not otherwise specified (NOS) in nine cases; EBV‐positive DLBCL, NOS in two cases; and T‐cell/histiocyte‐rich large B‐cell lymphoma in two cases. Five cases of DLBCLs were not re‐classified because their EBV status was unknown. The Hans algorithm, which uses a combination of staining for CD10, BCL6, and MUM1, was used to classify the DLBCLs into the germinal center B‐cell (GCB) subtype for three cases and the non‐GCB subtype for nine cases.

**Conclusion:**

These results indicate that DLBCL tends to occur late in pSS cases and is mainly related to the non‐GCB subtype of DLBCL.

## 1 INTRODUCTION

Sjögren's syndrome (SS) is a chronic autoimmune disease which damages the exocrine glands, notably the salivary and lacrimal glands. It is histologically characterized by lymphocytic infiltration and destruction of the exocrine glands and can occur alone (primary SS; pSS) or in association with other autoimmune rheumatic diseases. Recent research has suggested that SS may be associated with the development of diffuse large B‐cell lymphoma (DLBCL), with the relative risk of DLBCL development in patients with pSS ranging from 2.0 to 6.57.^1^, ^2^ The broad variability in this risk can be attributed to differences in the SS diagnosis criteria and follow‐up duration. Nevertheless, DLBCL is an aggressive and heterogeneous lymphoid tumor which affects the quality of life and prognosis of patients with pSS. Morphological, immunophenotypic, molecular, and clinical studies have subdivided DLBCL into morphological variants, molecular subtypes, and distinct disease entities that have different prognoses and treatment choices.^3^ Cases involving DLBCL that do not fulfill the criteria for the specific disease entities are referred to as DLBCL, not otherwise specified (NOS). However, there are few studies that have focused on characterizing DLBCL which occurs in patients with underlying pSS, and the present study aimed to describe the characteristics of DLBCL in a group of consecutive patients with confirmed diagnoses of pSS.

## 2 PATIENTS AND METHODS

Between June 1997 and June 2019, this retrospective study identified 2021 adult patients (>18 years old) who were diagnosed with SS at the VA Nasonova Research Institute of Rheumatology (Moscow, Russia). These patients included 25 with DLBCL. We excluded seven patients because they had SS with concomitant rheumatoid arthritis (RA), systemic lupus erythematosus, or systemic scleroderma. The medical records for the remaining 18 cases were reviewed to obtain information regarding age, gender, clinical presentation, laboratory findings, the time from the manifestation of SS to the diagnosis of DLBCL, relevant imaging studies, lymphoma treatment, follow‐up interval, and outcome.

All patients underwent diagnostic testing for xerostomia (unstimulated whole saliva flow), xerophthalmia (Schirmer's test, tear break‐up time, and Rose Bengal staining), and lip minor salivary gland biopsy. Immunologic tests that included antinuclear antibodies were performed using indirect immunofluorescence and tissue cryostat sections of the liver. Rheumatoid factor was detected via nephelometry. Precipitating antibodies against the extractable nuclear antigens Ro/SS‐A and La/SS‐B were detected via enzyme‐linked immunosorbent assay. All subjects included in this study fulfilled the consensus criteria for pSS.^4^

The DLBCLs were identified via surgical biopsy of lymph nodes or extranodal tissues. The tissue specimens were fixed in 10% formalin, routinely processed, and embedded in paraffin. The original hematoxylin‐eosin‐stained slides were used in all cases. Immunohistochemical (IHC) tests were performed using the formalin‐fixed paraffin‐embedded tissues. The broad antibody panel involved the following antibodies used at the manufacturer‐recommended dilutions: anti‐CD2 (clone MRQ‐11, Cell Marque), CD3 (clone F7.2.38, Dako), CD5 (clone 4C7, Dako), CD7 (clone MRQ‐56, Cell Marque), CD15 (clone C3D1, Dako), CD20 (clone L26, Dako), CD30 (clone Ber‐H2, Dako), CD43 (clone MT1, Cell Marque), cyclin D1 (clone SP4, Cell Marque), BCL2 (clone 124, Cell Marque), BCL6 (clone EP278, Cell Marque), MUM1 (clone MRO‐8, Cell Marque), PAX5 (clone DAK‐Pax5, Dako), HGAL (clone MRQ‐49, Cell Marque), c‐MYC (clone EP121, Cell Marque), and Ki‐67 (clone SP6, Cell Marque). After dewaxing and heat‐induced antigen retrieval, the sections were stained by using an Autostainer Link 48 (Dako) according to the manufacturer's instructions. The expressions of HGAL, CD10, BCL6, and MUM1 were considered positive if ≥30% of the tumor cells exhibited positive staining.^5^ The expression of BCL2 was considered positive if ≥50% of the tumor cells exhibited positive staining.^3^ The expression of MYC was considered positive if ≥40% of the tumor cell nuclei exhibited positive staining.^3^

In situ hybridization was performed on formalin‐fixed paraffin sections to detect Epstein‐Barr virus (EBV) in lymphoma tissue in 13 cases; five cases were not evaluated due to the lack of or poor quality of samples. To detect EBV‐encoded small nuclear ribonucleic acid (EBER), a mixture of fluorescein‐conjugated oligodeoxyribonucleotides complementary to the two EBERs (EBER; Dako) was used.

In cases 1, 13, and 15, a c‐MYC/8q24 gene locus translocation test was performed on paraffin sections using fluorescence in situ hybridization (FISH) with the LSI c‐MYC Break Apart Probe (Abbott) and the LSI c‐MYC/IgH Dual Fusion Translocation Probe.

All patients were staged according to the Ann Arbor staging system and stratified according to the original International Prognostic Index (IPI).^6^ The DLBCLs were also re‐classified according to the 2016 WHO classification of tumors of hematopoietic and lymphoid tissues.^3^ According to the revised response criteria for malignant lymphomas, overall survival (OS) was defined as the time from the diagnosis until death because of any cause.^7^

### 2.1 Statistical analysis

Categorical variables are reported as number (%) and continuous variables are reported as median (range). OS was estimated using the Kaplan‐Meier method (time from the lymphoma diagnosis until the date of death or the last follow‐up) and was compared using the log‐rank test. All research was performed in accordance with the relevant guidelines.

## 3 RESULTS

Between June 1997 and June 2019, we identified 18 eligible patients with pSS‐related DLBCL. The patients’ clinical characteristics, immunological data, and lymphoma risk factors are shown in Table 1. All 18 patients were women, and the median age at the onset of the initial pSS symptoms was 34 years (range: 20‐61 years). The median time from the onset of the initial pSS symptoms to the DLBCL diagnosis was 20.5 years (range: 6‐38 years), and the median time from the pSS diagnosis to the DLBCL diagnosis was 14 years (range: 1‐27 years). The median age at the DLBCL diagnosis was 55 years (range: 39‐83 years). A monoclonal component in serum and/or urine was detected in 47% of the tested patients (8/17), based on trace secretion. Serum specimens from three cases contained paraprotein immunoglobulin M (IgM) kappa. Urine specimens from four patients contained Bens‐Jones protein, kappa type. One patient had a combination of paraprotein IgM kappa in the serum specimen and Bens‐Jones protein, kappa type in the urine specimen. Cryoglobulinemia was observed in 82% of the tested cases (14/17), and a decreased level of complement C4 was observed in 43% of the tested patients (3/7).

**Table 1:** Baseline characteristics, immunological data, and lymphoma risk factors of the 18 patients with primary Sjögren's syndrome and diffuse large B‐cell lymphoma

Case no.	Gender	Age (y) at initial SS symptoms	Anti‐SSA/Ro	Anti‐SSB/La	RF	ANA	Cryoglobulinemia	Lymphopenia^a^	Low C4 complement	Monoclonal component
1	F	20	−	−	+	+	−	−	−	−
2	F	30	NA	NA	+	+	+	+	NA	−
3	F	35	NA	NA	+	+	−	−	NA	−
4	F	20	+	−	+	+	+	−	NA	−
5	F	61	NA	NA	+	+	+	+	NA	−
6	F	46	+	+	+	+	+	−	−	−
7	F	21	NA	NA	+	+	+	–	NA	+ (BJκ)
8	F	29	NA	NA	+	+	+	+	NA	−
9	F	33	+	+	−	+	NA	NA	NA	NA
10	F	47	NA	NA	+	+	+	NA	NA	+ (IgMκ)
11	F	23	NA	NA	+	+	+	NA	NA	+ (BJκ)
12	F	52	NA	NA	+	+	+	−	NA	+ (BJκ)
13	F	55	+	−	+	+	+	–	−	−
14	F	49	−	−	+	+	−	+	−	+ (BJκ)
15	F	40	+	−	+	+	+	+	+	+ (IgMκ + BJκ)
16	F	21	+	−	+	+	+	+	+	+ (IgMκ)
17	F	21	+	+	+	+	+	NA	NA	−
18	F	43	−	−	+	+	+	NA	+	+ (IgMκ)
Total	100%	Median age: 34 y (range: 20‐61)	70% (7/10)	30% (3/10)	94% (17/18)	100% (18/18)	82% (14/17)	46% (6/13)	43% (3/7)	47% (8/17)

Abbreviations: −, negative; +, positive; ANA, antinuclear antibodies; BJ, Bence Jones protein; Ig, immunoglobulin; NA, not available; RF, rheumatoid factor.

a Lymphocyte count < 1000/μL at pSS diagnosis.

The pathological findings of DLBCLs are shown in Table 2. Morphological evaluations revealed a centroblastic variant of DLBCL in cases 2, 4‐12, and 15, while an anaplastic variant of DLBCL was observed in case 3. Cases 16‐18 had a limited number of scattered large B cells (CD20+, CD15−, CD30−) embedded in a background of abundant histiocytes and CD3 + and CD5 + T cells. Case 16 was initially misinterpreted as having T‐cell/histiocyte‐rich large B‐cell lymphoma (THRLBCL), but detection of EBV in large cells allowed us to regard this case as a polymorphic variant of EBV‐positive DLBCL. The histology and immunophenotype in cases 17 and 18 were consistent with THRLBCL. Cases 1, 13, and 14 had predominantly medium‐sized tumor cells. The lack of c‐MYC/8q24 rearrangements, as well as cyclin D1 and MYC expression in the tumor cells allowed for a diagnosis of DLBCL. The MYC protein was detected only in case 15. A FISH study of tumor tissue in this case revealed additional signal at the c‐MYC/8q24 gene locus and the lack of c‐MYC/8q24 rearrangements. The detection of EBV in tumor cells allowed us to classify this case as EBV‐positive DLBCL, NOS.

**Table 2:** Pathological findings from 18 DLBCL cases

Case no.	CD20	HGAL (>30%)	CD10 (>30%)	BCL6 (>30%)	MuM1 (>30%)	BCL2 (>50%)	MYC (>40%)	EBV	Subtype according to Hans algorithm	DLBCL type
1	**+**	**+**	**+**	**+**	**+**	**−**	**−**	**−**	GCB	DLBCL, NOS
2	**+**	**−**	**−**	**+**	**+**	**−**	**−**	**−**	Non‐GCB	DLBCL, NOS
3	**+**	**−**	**−**	**−**	**+**	**−**	**−**	**−**	Non‐GCB	DLBCL, NOS
4	+	**−**	**−**	**−**	+	**−**	**−**	**−**	Non‐GCB	DLBCL, NOS
5	+	**−**	**−**	+	+	+	**−**	**−**	Non‐GCB	DLBCL, NOS
6	+	**−**	**−**	+	+	**−**	NA	NA	Non‐GCB	DLBCL
7	+	NA	NA	NA	NA	NA	NA	NA	NA	DLBCL
8	+	**−**	**−**	**−**	**+**	**−**	**−**	**−**	Non‐GCB	DLBCL, NOS
9	+	**−**	**−**	**+**	**+**	**+**	**−**	**−**	Non‐GCB	DLBCL, NOS
10	+	**−**	**−**	**−**	**+**	**−**	**−**	**−**	Non‐GCB	DLBCL, NOS
11	+	NA	NA	NA	NA	NA	NA	NA	NA	DLBCL
12	+	NA	NA	NA	NA	NA	NA	NA	NA	DLBCL
13	+	NA	**−**	**+**	**−**	+	**−**	NA	GCB	DLBCL
14	+	−	**−**	**+**	**−**	+	**−**	−	GCB	DLBCL, NOS
15	+	**+**	**−**	**−**	**+**	+	**+**	**+**	Non‐GCB	EBV‐positive DLBCL, NOS; monomorphic
16^a^	+	NA	NA	NA	NA	NA	NA	**+**	NA	EBV‐positive DLBCL, NOS; polymorphic
17^a^	+	NA	NA	NA	NA	NA	NA	**−**	NA	THRLBCL
18^a^	+	NA	NA	NA	NA	NA	NA	**−**	NA	THRLBCL

Abbreviations: −, negative; +, positive; DLBCL, diffuse large B‐cell lymphoma; DLBCL, NOS, diffuse large B‐cell lymphoma, not otherwise specified; EBV, Epstein‐Barr virus; GCB, germinal center; NA, not available; THRLBCL, T‐cell/histiocyte‐rich large B‐cell lymphoma.

a Cases 16‐18 had a limited number of scattered tumor cells, making it difficult to calculate the percentage of positively stained cells.

Of 18 cases of DLBCLs, in three cases there were no samples for IHC study (cases 7, 11, and 12), another three cases had a limited number of scattered large cells (cases 16‐18), which interfered with calculating the percentage of positively stained tumor cells. The remaining 12 cases of DLBCLs, according to the Hans algorithm,^5^ were subdivided into the germinal center B‐cell (GCB) subtype for three cases and the non‐GCB subtype for nine cases. The HGAL staining results matched those predicted by the Hans model in nine of the 11 tested cases.

According to the 2016 WHO classification, the 13 cases were classified as: DLBCL, NOS (nine cases), EBV‐positive DLBCL, NOS (two cases), and THRLBCL (two cases). Five cases of DLBCLs were not re‐classified because their EBV status was unknown.

**Figure 1:**
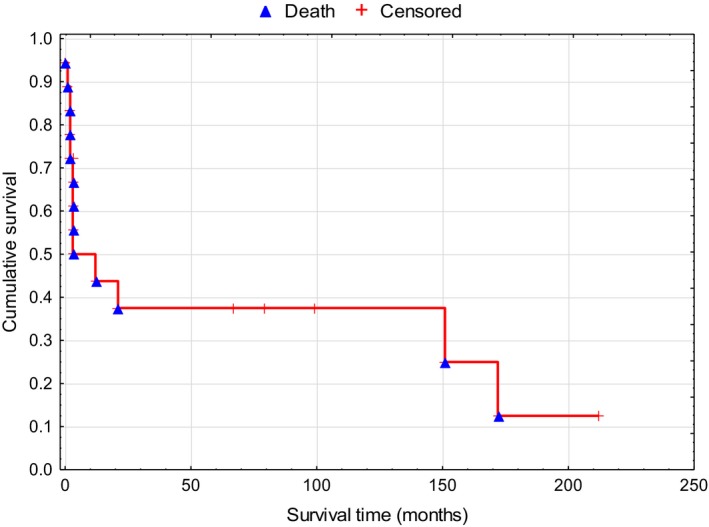
Overall survival among the 18 patients with primary Sjögren's syndrome and diffuse large B‐cell lymphoma

Based on the IPI, six patients were assigned to low and low/intermediate risk groups, while 12 patients were assigned to intermediate/high and high risk groups. Extranodal DLBCL lesions were detected in 14 patients, which most commonly involved the lungs (six patients) and stomach (two patients). Baseline clinical characteristics, treatments, and outcomes for the 18 pSS‐related DLBCLs are shown in Table 3. The average follow‐up period after the diagnosis of DLBCL was 44 months (range: 0‐200 months). The OS of the patients after the lymphoma diagnosis is shown in Figure 1. The median OS after the lymphoma diagnosis was 3 months (range: 0‐212 months), and the 5‐year OS rate after the lymphoma diagnosis was 37.5%.

**Table 3:** Clinical characteristics, treatments, and outcomes for the 18 pSS‐related DLBCLs

Case no.	Age (y) at DLBCL diagnosis	Time (y) from the onset of SS symptoms to the diagnosis of DLBCL	IPI	Lymphoma location	DLBCL treatment	Outcome/DLBCL evolution/survival from DLBCL diagnosis (mo)
1	52	32	Low/intermediate	Liver	R‐CHOP × 6	Alive/CR/87
2	59	19	Intermediate/high	Bone marrow, buccal mucosa, lymph nodes	NA	Dead/NA/3
3	53	18	Low/intermediate	Lymph nodes	CHOP × 6	Death from colon cancer/CR/172
4	48	38	Low	Lymph nodes	R‐CHOP × 6	Dead/CR/151
5	83	22	Intermediate/high	Lymph nodes	NA	Dead/DP/12
6	59	13	Low	Stomach	R‐CHOP × 6	Alive/CR/67
7	47	26	High	Brain, lung, lymph nodes	high doses of glucocorticosteroids	Death from cerebral edema/ postmortem diagnosis / 0
8	51	22	High	Parotid gland, lung, lymph nodes	NA	Dead/NA/3
9	39	6	High	Lung, stomach, lymph nodes	R‐CHOP × 2	Dead/NA/3
10	62	15	High	Lung, lymph nodes	NA	Dead/NA/2
11	45	22	Intermediate/high	Lung, spleen, lymph nodes	NA	Dead/NA/2
12	70	18	High	Lung, lymph nodes	CHOP × 3	Death from acute pulmonary embolism/CR/3
13	73	18	High	Retroperitoneal tumor with infiltration of the diaphragm crus, left psoas major muscle, and left kidney sinus	CHOP × 1	Dead/NA/2
14	73	24	Intermediate/high	Parotid gland, lymph nodes	R‐CHOP × 3	Alive/continuing therapy/4
15	61	20	Intermediate/high	Left mandibular fossa spreading anteriorly to the parotid area	R‐CHOP × 1	Dead/NA/1
16	42	21	Low/intermediate	Lymph nodes	VR‐CAP × 6/CR, relapse after 4 mo, ICE × 3	Dead/DP/21
17	43	22	Low	Amygdala, lymph nodes	CHOP × 6	Alive/CR/200
18	57	14	Intermediate/high	Spleen, posterolateral pharyngeal wall, lymph nodes	R‐CHOP × 6	Alive/CR/55

Abbreviations: CR, complete remission; DLBCL, diffuse large B‐cell lymphoma; DP, disease progression; EBV, Epstein‐Barr virus; ICE, ifosfamide, carboplatin, etoposide; IPI, International Prognostic Index; NA, not available; pSS, primary Sjögren's syndrome; R‐CHOP, rituximab, cyclophosphamide, doxorubicin, vincristine, and prednisone; VR‐CAP, bortezomib, rituximab, cyclophosphamide, doxorubicin, prednisone.

## 4 DISCUSSION

The main predictors of lymphoma development in patients with pSS are permanently enlarged salivary glands,^8^, ^9^, ^10^ palpable purpura,^9^, ^10^, ^11^ lymphadenopathies,^8^, ^10^ cryoglobulinaemia,^10^, ^12^ low complement levels (especially C4),^9^, ^10^, ^11^, ^13^ a monoclonal component in the serum or urine,^8^ and lymphopenia.^11^, ^13^ However, these studies evaluated risk factors for the development of the entire group of non‐Hodgkin's lymphomas, and did not indicate which predictors could lead to the development of a specific variant of non‐Hodgkin's lymphoma. Given that the predominant variant of non‐Hodgkin's lymphoma in patients with pSS is marginal zone B‐cell lymphoma (MZL),^14^ these risk factors presumably predispose patients to the development of MZL. To the best of our knowledge, only the study by Baimpa et al determined that the presence of lymphocytopenia (<1000 cells/µL) at the pSS diagnosis was a risk factor for the development of DLBCL.^15^ In our cohort of patients with pSS‐related DLBCLs, lymphopenia was also detected in 43% of the tested patients (6/13) at the time of their pSS diagnosis.

After reviewing the relevant literature, we only identified 117 cases of SS‐related DLBCL that have been described in case reports or small case series.^2^, ^11^, ^13^, ^14^, ^15^, ^16^, ^17^, ^18^, ^19^, ^20^, ^21^, ^22^, ^23^, ^24^, ^25^, ^26^, ^27^, ^28^, ^29^, ^30^, ^31^, ^32^ This very small sample size is likely related to the low prevalence of pSS in the general population, which is estimated to be 0.2%–1.4%,^33^ combined with the estimated incidence of DLBCL, which is 7/100 000 population.^34^ For example, Chiu et al identified only nine cases of DLBCL in 16 396 patients with pSS,^31^ while Smedby et al conducted a pooled analysis using the InterLymph Consortium database and identified only eight cases of DLBCL in 8176 patients with pSS.^2^ An analysis of the literature, as well as our results, indicate that DLBCL is the predominant variant among large B‐cell lymphomas in patients with pSS, and only sporadic cases have involved other specific entities of large B‐cell lymphomas in patients with pSS: one case of THRLBCL,^25^ two cases of primary cutaneous DLBCL, leg type,^13^, ^17^ one case of primary DLBCL of the central nervous system,^20^ one case of intravascular large B‐cell lymphoma,^24^ one case of lymphomatoid granulomatosis,^32^ and three cases of EBV‐positive DLBCL, NOS.^26^, ^28^, ^32^

According to Vasaitis, EBV was detected in the lymphoma tissue in 22% of investigated cases of pSS‐related DLBCLs.^32^ In our cohort, EBV was detected in the DLBCL tissue for 15.4% of the cases. Baecklund et al analyzed a large group of patients with RA and DLBCL, and detected EBV in 8.6% of the RA‐related DLBCL cases.^35^ The results of these studies and our data indicate that the prevalence of EBV‐positive DLBCL, NOS is higher among DLBCLs in patients with autoimmune diseases, relative to that in the general population.^3^ This suggests that the EBV may play a role in the pathogenesis of DLBCLs associated with autoimmune diseases.

In our study, the DLBCL patients were younger at their diagnosis (median age: 55 years), relative to other studies (median age: 67 years).^11^, ^32^ Interestingly, pSS‐related DLBCLs tend to arise in individuals with longstanding pSS, and Theander et al reported that the median time from the onset of sicca symptoms to the DLBCL diagnosis was 17 years, and the median time from the pSS diagnosis until DLBCL development was 10 years.^11^ Vasaitis reported similar results, with a median time of 18.5 years from the onset of sicca symptoms to the DLBCL diagnosis and a median time of 9 years from the pSS diagnosis until DLBCL development.^32^ Similarly, we observed that the median time from the initial symptoms of pSS to the DLBCL diagnosis was 20.5 years, and the median time from the pSS diagnosis until the DLBCL diagnosis was 14 years. Interestingly, Baecklund et al reported that the duration of RA before the diagnosis of DLBCL was also 20 years.^35^

The algorithm of Hans et al uses a combination of three immunostaining markers (CD10, BCL6, and MUM1) and allows for DLBCL to be classified into two subsets with different pathologies, treatment outcomes, and prognoses: the GCB subtype with a better prognosis and the non‐GCB subtype with a worse prognosis.^5^, ^36^, ^37^, ^38^ Unlike the findings of Vasaitis,^32^ who reported similar proportions of the non‐GCB (13 cases) and GCB (13 cases) subtypes among pSS‐related DLBCLs, our group observed a significant predominance of the non‐GCB subtype (9 cases), relative to the GCB subtype (three cases) of DLBCLs. Kojima et al have also reported that all nine of their patients with RA‐related DLBCLs had the non‐GCB subtype,^39^ which also agrees with the findings of Baecklund et al, who reported a statistically significant predominance of the non‐GCB subtype (70% of 139 RA‐related DLBCLs).^35^

However, comparison of this IHC model with the gold‐standard gene expression profiling revealed a 20% misclassification rate,^5^ has highlighted the need for additional IHC markers to increase the model's predictive value. Human germinal center‐associated lymphoma (HGAL) is a B‐cell specific marker the expression of which is observed in the cytoplasm of normal germinal center B cells and lymphomas of GCB derivation.^40^ Gualco et al reported that the inclusion of HGAL in the Hans immunohistological algorithm improved the detection of the GCB subtype of DLBCL.^41^ In our study, the HGAL staining results matched those predicted by the Hans model in 82% (9/11) of cases.

In the group of pSS‐related DLBCLs that were analyzed by Vasaitis, the overall median survivals were similar between the non‐GCB and GCB subtypes.^32^ However, given the small sample of patients with the GCB subtype, we could not assess the difference in survival between the non‐GCB and GCB subtypes. Nevertheless, the median survival time after the lymphoma diagnosis in our group was only 3 months, which was significantly shorter than the result from the Vasaitis cohort (6 years).^32^ The short survival in our cohort may be related to several reasons. First, many DLBCL patients were diagnosed in the early 2000s, when the therapeutic options did not include rituximab for DLBCL treatment. In these patients, the non‐GCB subtype of DLBCL according to the IHC‐based Hans algorithm was associated with significantly poorer OS.^42^ Second, approximately two‐thirds of our cases belonged to the medium/high and high risk groups based on the IPI, which have relatively poor outcomes. Third, most of the deaths in our cohort were observed during chemotherapy. Since chemotherapy was administered in an outpatient setting for most patients, we assume a lack of timely doctor‐patient feedback which could lead to an untimely initiation of the therapy for febrile neutropenia.

Rheumatologists treating pSS patients should remember that DLBCL can manifest under several clinical masks. For example, case 1 involved isolated hepatic lymphoma which could be misdiagnosed as hepatocellular carcinoma or liver metastasis. In other instances, the lymphoma manifested as peripheral paralysis of the facial nerve (case 15) or diabetes insipidus (case 7).

The mechanisms underlying the pathogenesis of pSS‐related DLBCL remain unclear, although the increased prevalence of the non‐GCB DLBCL subtype in our cohort is consistent with the hypothesis of chronic B‐cell stimulation and antigenic drive. However, given that a proportion of DLBCLs were of the GCB subtype, the malignant transformation is likely also related to other pathways. In conclusion, we found that pSS‐related DLBCLs arose relatively late in the course of pSS, with a median interval of 20.5 years between the onset of the pSS symptoms and the lymphoma diagnosis. The non‐GCB subtype of DLBCL was also prevalent in our cohort, and these characteristics seem similar to those of RA‐related DLBCL.

## CONFLICT OF INTEREST

The authors declare they have no conflicts of interest regarding the publication of this study.

## AUTHOR CONTRIBUTIONS

VRG acquired and analyzed the data, wrote the paper, and revised the paper and formulated conclusions with NAP and VIV. NAP analyzed the data. VIV acquired and analyzed the data. All authors approved the final manuscript and agree to be accountable for all aspects of the work.

## COMPLIANCE WITH ETHICAL STANDARDS

Ethical approval for the study's retrospective protocol was obtained from the VA Nasonova Research Institute of Rheumatology Ethics Committee. Patients provided informed consent for the collection and analysis of their data and specimens.
